# Cognition‐Associated Changes in Retinal Thickness Relate to Limbic and Temporal Cortical Atrophy in Parkinson's Disease

**DOI:** 10.1002/brb3.70509

**Published:** 2025-05-05

**Authors:** Kerstin Schweyer, Tobias Mantel, Benjamin Knier, Lilian Aly, Jan S. Kirschke, Tobias Meindl, Bernhard Haslinger

**Affiliations:** ^1^ Department of Neurology, Klinikum rechts der Isar, TUM School of Medicine and Health Technical University of Munich Munich Germany; ^2^ TUM Neuroimaging Center (TUM‐NIC), Klinikum rechts der Isar, TUM School of Medicine and Health Technical University of Munich Munich Germany; ^3^ Department of Neurology Diakoneo Diak Klinikum Schwäbisch Hall Schwäbisch Hall Germany; ^4^ Department of Diagnostic and Interventional Neuroradiology, Klinikum rechts der Isar, TUM School of Medicine and Health Technical University of Munich Munich Germany

**Keywords:** atrophy, cognition, cognitive dysfunction, gray matter, magnetic resonance imaging, Parkinson disease

## Abstract

**Background:**

Research links retinal changes to cognitive decline in Parkinson's disease (PD), paralleling findings in Alzheimer's, raising questions about specific cortical patterns of cognition‐related retinal abnormalities in PD.

**Objective:**

The study aimed to explore whether retinal thinning linked to cognitive decline could act as a potential biomarker for cerebral atrophy in PD.

**Methods:**

Twenty seven patients with PD underwent cognitive and neurological assessments, along with retinal imaging using OCT and cerebral imaging using structural MRI. After identifying abnormal retinal layers associated with cognitive dysfunction through partial correlation analyses controlling for age‐related effects, associations between these retinal layers and the parcellated cerebral gray matter were assessed using multiple comparison‐corrected partial correlation analyses adjusted for age and gender.

**Results:**

Significant positive correlations were found between cognitive impairment measured by MoCA and specific retinal layers (IPL, GCL, and RNFL). Of these, strong associations were observed between the IPL and GCL and cortical thickness in brain the temporal lobe and limbic cortex, with more detailed further analysis showing significant correlations particularly within the middle and posterior cingulate cortex in the limbic cortex and the middle and superior temporal gyrus in the temporal lobe.

**Conclusion:**

Correlations between retinal thinning, cognitive decline, and specific patterns of cortical atrophy in PD support a potential of retinal measurements as a biomarker for cognitive impairment linked to cerebral neurodegeneration.

## Graphical abstract

Twenty‐seven Parkinson's disease patients underwent cognitive assessment (MoCA), retinal OCT imaging and brain MRI. Thinning in specific retinal layers (IPL, GCL) correlated both with cognitive decline and thinning of temporal and limbic cortices. This supports retinal imaging as a potential biomarker for detecting neurodegeneration‐related cognitive decline in the disease.

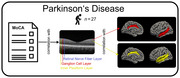

## 1 Background

Parkinson's disease (PD) is a neurodegenerative disease presenting with the cardinal motor symptoms of rigidity, resting tremor, and bradykinesia. Aside from motor symptoms, numerous non‐motor symptoms occur, some of them anticipating the onset of motor symptoms (Goldman and Postuma 2014). Those symptoms are thought to be caused by a spread of the underlying α‐synuclein pathology outside the nigro‐striatal pathway in the brain (Adler and Beach 2016). Interestingly, beyond cerebral gray matter, central α‐synuclein pathology was also found in the dopaminergic neurons of the retina (Bodis‐Wollner et al. 2014; Marrocco et al. 2020; Veys et al. 2019), and alterations in retinal architecture were observed in PD patients (Albrecht et al. 2012; Garcia‐Martin et al. 2014; Hajee et al. 2009).

Cognitive impairment is one of the most disabling and important non‐motor symptoms in PD (Aarsland et al. 2021; Leroi et al. 2012). An association of retinal thinning with cognitive decline was found not only in PD (Murueta‐Goyena et al. 2021; Sung et al. 2019; Zhang et al. 2021) but also in other dementias such as Alzheimer's disease (Kim and Kang 2019). In the latter, studies have shown associations of retinal thinning with specific cortical atrophy patterns (den Haan et al. 2018, 2019). Yet to date, it remains an open question if cognition‐associated retinal changes in PD are also accompanied by characteristic cortical atrophy patterns.

We therefore intended to investigate if retinal thinning associated with cognitive decline could serve as a biomarker for cerebral atrophy in PD. Therefore, we studied the correlation of retinal parameters measured by optical coherence tomography (OCT) and cognition in PD patients, followed up with a correlation of these retinal parameters with morphometric neuroimaging markers for cerebral atrophy.

## 2 Methods

### 2.1 Participants

We included 27 patients with PD who regularly presented to our outpatient movement disorders clinic between 2019 and 2020, diagnosed with clinically definite (17/27) or probable (10/27) PD following the 2015 Movement Disorders Society criteria (Postuma et al. 2015). Diagnosis was established by an experienced neurologist with movement disorders expertise. Patients with a medical history of ophthalmological diseases (glaucoma, membranous, age‐related macular degeneration, diabetic retinopathy, epiretinal membrane, and retinal vein or artery occlusion), diabetes mellitus, and uncontrolled arterial hypertension were not included in the study. Data acquisition was approved by the local ethics committee (https://www.ek‐med‐muenchen.de/) and written informed consents according to the Declaration of Helsinki were obtained from the participants.

### 2.2 Clinical Assessment

All patients underwent a structured neurological clinical exam and cognitive testing. Cognition was evaluated using the Montreal Cognitive Assessment (MoCA), which is recommended to assess cognitive impairment in PD (Dalrymple‐Alford et al. 2010). In addition, disease stage (Hoehn and Yahr (H&Y) scale), and motor impairment (UPDRS III) were assessed in the ON‐state.

### 2.3 OCT Acquisition and Analysis

OCT images were acquired using a spectral domain OCT device (Spectralis OCT2; Heidelberg Engineering) as described elsewhere (Knier et al. 2016). A signal strength greater than 15 dB was considered sufficient for retinal segmentation and volume assessment, and the OSCAR‐IB criteria were followed for thorough OCT quality control (Tewarie et al. 2012). Retinal segmentation of B‐scans was conducted automatically using the inbuilt Eye Explorer software (Eye Explorer, v2.5.4.). Afterward, segmentations were manually checked and corrected in a blinded manner if necessary. Evaluation of the peripapillary retinal nerve fiber layer (pRNFL) was performed using a 3.4‐mm ring scan centered on the optic nerve head (automatic real time: 100). The macular area underwent 61 vertical B‐scans (scanning angle, 30° × 25°) focusing on the fovea centralis. The macular retinal nerve fiber layer (RNFL), the ganglion cell layer (GCL), the inner plexiform layer (IPL), the inner nuclear layer (INL), the outer plexiform layer (OPL), the outer nuclear layer (ONL), and the retinal thickness (RT) were segmented for further study. Layer volumes were calculated by the software's segmentation algorithm (6‐mm‐diameter circle around the fovea).

### 2.4 MRI Acquisition and Analysis

High‐resolution structural neuroimaging (T1, FLAIR; acquisition parameters are provided in Table S1) was acquired on a Philips Achieva 3 Tesla MRI scanner within 3 months of OCT acquisition as part of a prospective longitudinal multimodal neuroimaging cohort study. Each scan underwent a qualitative neuroradiological, and a quantitative noise‐to‐contrast ratio‐based quality control (as implemented in CAT12r1152) (Dahnke et al. 2013). T1 scans were skull‐stripped and introduced into FreeSurfer v6.0 for automated reconstruction of the cortical surfaces and segmentation of the deep gray matter structures, using standard parameters and the FLAIR image for improved pial surface reconstruction (Fischl 2012). Cortical surfaces were parcellated using the Destrieux atlas, and cortical thickness was extracted for each atlas ROI (Destrieux et al. 2010). For the deep gray matter structures, gray matter volumes were extracted.

### 2.5 Statistical Analysis

Statistical analyses were conducted using SPSS29 and MatlabR2021a. As we did not assume lateralized ocular involvement in the disease and the visual information from one eye is processed in both hemispheres, the retinal and neuroimaging parameters were averaged across eyes and hemispheres, respectively, for all statistical analyses. We first identified those segmented retinal layers reported abnormal in PD (Table 1) (Alves et al. 2023) that showed a significant association with cognitive dysfunction through partial correlation analyses controlling for age‐related effects on both retinal parameters and MoCA performance (*p* < 0.05). Then, retinal layers showing such a relationship were tested for significant associations with regional cerebral gray matter (cortical thickness and subcortical volumes adjusted for the total intracranial volumes), applying partial correlation analyses controlling for the influence of age and gender on both retinal and neuroimaging measures. We primarily identified significant associations with six predefined major cerebral domains (frontal, parietal, occipital, temporal, limbic cortex and subcortex, see Table S2 for details; p × 6 for a p_FWE(Bonf)_ < 0.05; Table 2). Significant associations were further detailed in a multiple comparison‐corrected follow‐up investigation of the cortical areas within these domains (p_FDR_ < 0.05; Table 2).

**TABLE 1:** Clinical and OCT characteristics.

Age (mean ± SD)	62.3 ± 11.4
Sex (m/f)	20/7
Hoehn and Yahr (ON) (median, IQR)	2, 0.5
Disease duration, months (mean ± SD, [95% CI])	52.0 ± 43.8 [31.4 72.5]
UPDRS III (ON) (mean ± SD, [95% CI])	17.0 ± 7.1 [13.6 20.3]
Levodopa (equivalent) dose, mg (mean ± SD)	456.8 ± 259.7
MoCA (mean ± SD, [95% CI])	25.87 ± 2.97 [23.9 26.8]
pRNFL, µm (mean ± SD)	95.43, 13.86
RT, µm (mean ± SD)	273.38, 25.16
RNFL, mm^3^ (mean ± SD)	0.87, 0.10
GCL, mm^3^ (mean ± SD)	1,01, 0.12
IPL, mm^3^ (mean ± SD)	0.87, 0.10
INL, mm^3^ (mean ± SD)	0.94, 0.08
OPL, mm^3^ (mean ± SD)	0.79, 0.08
ONL, mm^3^ (mean ± SD)	1.65, 0.18

Abbreviations: GCL, ganglion cell layer; INL, inner nuclear layer; IPL, inner plexiform layer; MoCA, Montreal Cognitive Assessment; OPL, outer plexiform layer; ONL, outer nuclear layer; pRNFL, peripapillary retinal nerve fiber layer; RT, retinal thickness; RNFL, macular retinal nerve fiber layer; UPDRS, Unified Parkinson's Disease Rating Scale.

**TABLE 2:** Correlation analyses results.

OCT layer	IPL		GCL		RNFL	
	*p*_adj_	*r*	*p*_adj_	*r*	*p*_adj_	*r*
**Frontal**	0.782	0.325	1	0.268	1	0.033
**Parietal**	0.369	0.396	0.879	0.312	1	0.151
**Occipital**	0.101	0.493	0.204	0.444	1	0.173
**Temporal**	**0.005**	**0.648**	**0.030**	**0.565**	0.875	0.313
** Subregions**						
Superior temporal gyrus (lateral)	0.037	0.481				
Middle temporal gyrus	0.009	0.625	0.0268	0.553		
Inferior temporal sulcus	0.012	0.595	0.0268	0.559		
Superior temporal sulcus	0.008	0.659	0.0140	0.637		
Occipito‐temporal sulcus (lateral)	0.037	0.482				
**Limbic**	**0.022**	**0.580**	**0.013**	**0** **.607**	0.210	0.441
** Subregions**						
Middle‐anterior cingulate gyrus and sulcus	0.015	0.617	0.002	0.694		
Middle‐posterior cingulate gyrus and sulcus	0.045	0.482	0.040	0.508		
Posterior‐dorsal cingulate gyrus	0.037	0.537	0.032	0.545		
**Subcortex (cerebellum, subcortical nuclei)**	0.552	0.360	0.479	0.373	0.070	0.516

Abbreviations: GCL, ganglion cell layer; IPL, inner plexiform layer; *p*_adj_, adjusted *p* value (significance at p_FWE(Bonf)_ < 0.05 adjusted for the six predefined cerebral domains is highlighted in bold; significance at p_FDR_ < 0.05 for follow‐up analyses of subregions); *r* = correlation coefficient; RNFL, macular retinal nerve fiber layer.

## 3 Results

### 3.1 Demographics

Included PD patients showed mild‐to‐moderate disease‐related impairment and motor symptoms in the ON state (Table 1). The average MoCA score was on the threshold to cognitive impairment, with 44.4% fulfilling previously suggested screening (< 26/30), and 18.5% the diagnostic (< 24/30) MoCA cut‐off scores for neurocognitive impairment in PD (Dalrymple‐Alford et al. 2010; Hu et al. 2014). MoCA was not significantly correlated with the degree of disease‐related motor impairment in the ON state (H&Y: *ρ* = −0.26, *p* = 0.21; UPDRS‐III: *r* = −0.08, *p* = 0.68).

### 3.2 Association of Retinal Thinning With Cognitive Impairment and Cortical Atrophy

**FIGURE 1:**
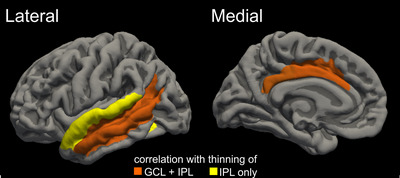
Areas with significant correlation between cortical thickness and IPL and GCL. Areas associated with thinning of both GCL and IPL are shown in orange, areas associated with thinning only in relation to IPL in yellow.

One‐sided partial correlation analyses (adjusted for age) showed a significant, moderate positive correlation of IPL (*r* = 0.44, *p* = 0.016), GCL (*r* = 0.44; *p* = 0.016), and RNFL (*r* = 0.44; *p* = 0.017) volume with cognitive function by MoCA scores. There was no correlation of relevance of IPL, GCL, or RNFL with sex (*r* values −0.037/−0.044/−0.007, *p*‐values > 0.83). In relation to the cerebral gray matter, the IPL and GCL showed strong correlations with the cortical thickness of the temporal lobe (GCL: *r* = 0.57, p_FWE_ = 0.030; IPL: *r* = 0.65, p_FWE_ = 0.005) and the limbic cortex (GCL: *r* = 0.61, p_FWE_ = 0.013; IPL: *r* = 0.58, p_FWE_ = 0.022), whereas no lobar correlations were observed with the RNFL. The follow‐up analysis within the limbic cortex indicated significant correlations of both GCL and IPL, particularly with the middle and posterior cingulate cortex. Within the temporal lobe, the IPL showed significant correlations with the middle and superior temporal gyrus as well as adjacent sulci; the GCL showed an overlapping, yet less extensive correlation pattern with emphasis on the middle temporal gyrus and neighboring sulci (see Table 2 and Figure 1 for details).

## 4 Discussion

Our study shows that retinal thinning in PD is associated with a decline in cognitive function, and is linked to cortical atrophy of a mainly temporal and limbic pattern. Although the retina has been studied extensively in PD, to our knowledge, our study is the first one to investigate associations of regional cerebral (cortical) atrophy with retinal atrophy in PD. Previous studies on associations of retinal and cerebral neurodegeneration focused mainly on atrophy of subcortical structures, for which they showed associations with retinal atrophy (Ahn et al. 2018; Sung et al. 2019).

In our study, we found a significant correlation of cognitive function with the thickness of the IPL and the GCL, retinal layers that have been shown to contain dopaminergic neurons and where α‐synuclein accumulates in animal models (Beach et al. 2014; Veys et al. 2019). Overall, our findings are in line with other work that observed association of GCL/IPL or RNFL thickness with reduced cognitive functioning (Murueta‐Goyena et al. 2021; Sung et al. 2019; Zhang et al. 2021), and of GCL/IPL thickness with PD dementia risk scores (Leyland et al. 2020). Thinning of the inner retinal layers, which include the RNFL, GLC, and IPL, is hypothesized to result from structural remodeling following dopamine deficiency due to the loss and dysfunction of dopaminergic amacrine cells in the inner retinal layers, which affects the morphology of melanopsin‐containing retinal ganglion cells and synaptic circuitry, particularly in the inner plexiform layer (Adam et al. 2013; Ortuno‐Lizaran et al. 2018).

In our study, this cognitive function‐associated thinning of these layers was significantly correlated with cortical thinning of the middle and posterior cingulum and in lateral temporal cortices extending into the temporo‐occipital junction. Temporal and limbic cortical atrophy, together with frontal cortical changes, are the most commonly observed cortical abnormalities in morphometric MRI studies prior to the onset of overt cognitive symptoms in PD (Silbert and Kaye 2010). Similar patterns are observed in the development of Lewy body dementia, another α‐synucleinopathy that is primarily manifested by cognitive deficits (Kantarci et al. 2022). Consistently, the infiltration of frontal, temporal, and limbic cortices with α‐synuclein pathology is considered an important determinant of PD dementia and Lewy body dementia (Aarsland et al. 2021). Yet it must be emphasized that a relevant fraction of cognitively impaired PD patients, especially those that progress to overt PD dementia, display an Alzheimer‐type co‐pathology, and in these patients both pathologies synergistically promote cognitive decline (Irwin et al. 2013). Therefore, it remains to determine whether the observed cortical (and retinal) atrophy pattern results from α‐synuclein‐dominant neurodegeneration or rather mirrors a mixed‐pathology neuronal loss (Irwin et al. 2013; Moreno‐Ramos et al. 2013). In this regard, it seems interesting that previous studies in patients with dominant Alzheimer‐type pathology rather showed a predominant correlation of retinal changes with parietal and inferotemporal cortices (den Haan et al. 2018, 2019, 2018, 2019). Beyond pathology, it further remains an unresolved question if cortical neurodegeneration leads to retinal abnormality through retrograde trans‐synaptic degeneration, or if both retinal and cortical neuronal populations undergo a parallel disease‐related decline (Davis et al. 2016; Garzone et al. 2023). While inferences about causality in neuroimaging are ultimately limited, further research using longitudinal data or advanced imaging and analysis techniques (e.g., mediation analysis or others) may help to shed more light on this aspect in the future. In this context, the role of subcortical and brainstem nuclei along the visual pathways could also be considered in more detail.

Deteriorating cognitive function in PD is accompanied by increasing impairment in visual processing. In consideration of the fact that different domains of visual processing capacities seem to be affected, it has been suggested that both retinal and higher order neuronal dysfunction is involved (Weil et al. 2016). In one cross‐sectional evaluation, both GCL/IPL thickness and visual dysfunction conveyed an increased (algorithm‐derived) dementia risk in PD (Leyland et al. 2020). At the cerebral level, declining higher order visual abilities over time were correlated with posterior cerebral thinning (lateral temporal cortices, occipital and limbic areas) (Garcia‐Diaz et al. 2018). Visual deficits in PD were further shown to correlate with cortical atrophy and reduced medial temporal lobe connectivity of the posterior cingulate cortex (Lucas‐Jimenez et al. 2016). Besides higher order cognition and memory function, the cingulum has key attention functions (Leech and Sharp 2014). Visual attentional deficits contribute to an increased risk of cognitive decline (Uc et al. 2005) and also contribute to impairment in higher‐order visual abilities (Carrasco 2018; Weil et al. 2016). Overall, our results might therefore also be considered in support of a previously discussed possible relation of memory functions, visual impairment, and retinal thinning.

There are limitations to our study. First, the sample size was rather small, which also did not allow the characterization of potential differences between cognitive‐function defined subpopulations, including potential retinal thickness cut‐off values. Further, study participants did not receive an ophthalmological exam. While a thorough clinical history of ophthalmological diseases was taken, and OCT images were screened on signs of glaucoma or retinal membranes, we cannot exclude a non‐diagnosed eye disease affecting retinal parameters with absolute certainty.

To validate the specificity or retinal biomarkers in PD, longitudinal studies collecting MRI and OCT data over time as well as comparative studies in healthy volunteers and patients with other neurodegenerative forms of dementia (e.g., Alzheimer's disease, Lewy body dementia) should be performed in the future. This could allow drawing conclusions on the question on the specificity of abnormalities for an underlying α‐synuclein pathology compared to other neurodegeneration.

## 5 Conclusion

Overall, our study provides important evidence for the retina serving as a window to the brain in PD patients and supports the further exploration of OCT as a biomarker for cognitive impairment due to cerebral neurodegeneration. Further research on mechanisms connecting retinal and cortical degeneration in PD, and its usability as a potential early biomarker in presymptomatic patients are warranted.

## Author Contributions

**Kerstin Schweyer**: conceptualization, formal analysis, investigation, data curation, writing – original draft. **Tobias Mantel**: methodology, formal analysis, investigation, data curation, visualization, writing – original draft, writing – review and editing. **Benjamin Knier**: resources, writing – review and editing. **Lilian Aly**: writing – review and editing. **Jan S. Kirschke**: resources, writing – review and editing. **Tobias Meindl**: investigation, writing – review and editing, data curation. **Bernhard Haslinger**: resources, writing – review and editing, supervision.

## Ethics Statement

Data acquisition was approved by the local ethics committee (Ethikkommission der TU München, https://www.ek‐med‐muenchen.de/) and written informed consents according to the Declaration of Helsinki were obtained from the participants.

## Conflicts of Interest

Benjamin Knier reports a relationship with Heidelberg Engineering GmbH that includes: speaking and lecture fees and travel reimbursement. The other authors declare no conflicts of interest.

### Peer Review

The peer review history for this article is available at https://publons.com/publon/10.1002/brb3.70509

## Supporting information



Supporting Information

## Data Availability

The datasets used and/or analyzed during the current study are available from the corresponding author on reasonable request.
