# Supplementary material for: Cognition‐Associated Changes in Retinal Thickness Relate to Limbic and Temporal Cortical Atrophy in Parkinson's Disease
Source: Brain Behav. 2025 May 5;15(5):e70509. doi: 10.1002/brb3.70509 (PMC12050648; doi:10.1002/brb3.70509)
Supplement: Supplementary file 1 — Supporting Information [file BRB3-15-e70509-s001.docx]

**Supplementary Information to**

**Cognition-associated changes in retinal thickness relate to limbic and temporal cortical atrophy in Parkinson’s disease**

**Tab. s-1: Imaging parameters**

| Sequences | Direction of acquisition | Voxel-Size [mm³] | TE [ms] | TR [ms] | T IR [ms] | Duration [s] |
| --- | --- | --- | --- | --- | --- | --- |
| T1 | sagittal | 1x1x1 | 4 | 9 | 1000 | 365 |
| FLAIR | sagittal | 1x1x1 | 290 | 4800 | 1650 | 235 |

**Tab. s-2: Predefined ROIs in each lobe**

| **Frontal** | G.S_frontomargin, G.S_transv_frontopol, G_front_inf.Opercular, G_front_inf.Orbital, G_front_inf.Triangul, G_front_middle, G_front_sup, G_orbital, G_precentral, G_rectus, Lat_Fis.ant.Horizont, Lat_Fis.ant.Vertical, Lat_Fis.post, S_central, S_front_inf, S_front_middle, S_front_sup, S_orbital_lateral, S_orbital_med.olfact, S_orbital.H_Shaped, S_precentral.inf.part, S_precentral.sup.part, S_suborbital |
| --- | --- |
| **Parietal** | G.S_paracentral, G.S_subcentral, G_pariet_inf.Angular, G_pariet_inf.Supramar, G_parietal_sup, G_postcentral, G_precuneus, S_interm_prim.Jensen, S_intrapariet.P_trans, S_postcentral, S_subparietal |
| **Occipital** | G.S_occipital_inf, G_cuneus, G_occipital_middle, G_occipital_sup, G_oc.temp_med.Lingual, Pole_occipital, S_calcarine, S_collat_transv_post, S_oc_middle.Lunatus, S_oc_sup.transversal, S_occipital_ant, S_oc.temp_med.Lingual, S_parieto_occipital |
| **Temporal** | G_oc.temp_lat.fusifor, G_temp_sup.G_T_transv, G_temp_sup.Lateral, G_temp_sup.Plan_polar, G_temp_sup.Plan_tempo, G_temporal_inf, G_temporal_middle, Pole_temporal, S_collat_transv_ant, S_oc.temp_lat, S_temporal_inf, S_temporal_sup, S_temporal_transverse |
| **Limbic** | G.S_cingul.Ant, G.S_cingul.Mid.Ant, G.S_cingul.Mid.Post, G_cingul.Post.dorsal, G_cingul.Post.ventral, G_oc.temp_med.Parahip, G_subcallosal, S_cingul.Marginalis, S_pericallosal, |
| **Subcortex** | cerebellum, thalamus, caudate, putamen, pallidum, hippocampus, amygdala, accumbens, ventral DC |
